# Neuroendocrine tumors: A tertiary care hospital study on clinicopathology features and prognosis

**DOI:** 10.6026/973206300214414

**Published:** 2025-12-15

**Authors:** Sreedevi Jakka, Radhika Narayan, Anil Prasad, Minakshi Mishra, Amitabh Kumar Upadhyay

**Affiliations:** 1 Department of Pathology, Tata Main Hospital and Manipal Tata Medical College, Jamshedpur, Jharkhand, India; 2 Department of Medical Oncology, Tata Main Hospital, Jamshedpur, Jharkhand, India

**Keywords:** Neuroendocrine tumors, immunohistochemistry, Ki-67 index, tumor grading, survival analysis

## Abstract

Neuroendocrine tumors (NETs) are rare, heterogeneous malignancies with variable progression and diverse clinical manifestations. Therefore, it is of interest to analyze 12 histologically and immunohistochemically confirmed NET cases diagnosed between 2021 and 2025. Biopsies were obtained from primary or metastatic sites and tumor grading was determined using histomorphology and Ki-67 index. The median age at diagnosis was 60 years (range 17-87), with a male predominance (M:F = 1.4:1). The stomach was the most common primary site (33%). Seven patients (59%) had nonfunctional symptoms, while five (41%) presented with functional manifestations such as hypoglycemia, abdominal distension and diarrhea. Metastatic disease was noted in 33% of cases. Chemotherapy was administered in two grade III NETs. Overall survival was 100% in grades I and II, whereas grade III had a mean survival of 15 months. NETs require precise histopathologic and immunohistochemical differentiation, particularly in high-grade forms, to enable timely diagnosis and improved management strategies.

## Background:

Neuroendocrine tumors (NETs) are a rare and heterogeneous group of cancers characterized by variable growth rates, though they are often slow-growing. The incidence of NETs is about 2 per 100,000 populations, accounting for nearly 0.5% of all malignancies [1]. These tumors originate from amine-producing neuroendocrine cells that may arise in different parts of the body [2]. NETs and neuroendocrine carcinomas (NECs) are most frequently located in the gastrointestinal tract (over 60%), particularly in the midgut, foregut and hindgut regions. The lung represents the second most common site (>20%) [3]. Other less frequent sites include the head and neck, thymus, thyroid, breast, skin and genitourinary system [4], with some cases presenting from metastasis of an occult primary [4]. Based on the ability to produce active peptides and a host of hormones, the NETs are classified as being functional and nonfunctional [2], with the former often causing symptoms such as nausea, vomiting, abdominal pain, diarrhea and fatigue. The classification of Neuroendocrine neoplasms (NEN) has undergone several revisions in recent years, the most recent being the WHO Classification of Endocrine and Neuroendocrine neoplasms in 2022 [5]. According to the recent classification, neuronal paragangliomas are distinguished from epithelial neoplasms, which are divided into well-differentiated NETs and poorly differentiated NECs. NETs are well differentiated and preserve the molecular and morphological characteristics of neuroendocrine cells. They are graded as G1, G2 and G3 based on the proliferation index, determined by the mitotic count (number of dividing cells under the microscope (2 mm^2^)) and the Ki-67 labeling index assessed through immunohistochemistry [6]. In contrast, NECs are poorly differentiated epithelial tumors with marked atypia and abnormal molecular or genetic features while still expressing neuroendocrine markers. Therefore, it is of interest to evaluate the clinicopathological characteristics of NETs, classify them according to the WHO 2022 neuroendocrine neoplasms guidelines, assess treatment strategies and analyze overall survival.

## Materials and Methods:

## Study design and setting:

This prospective, observational study was conducted between January 2021 and December 2025 in a tertiary care oncology referral center. A total of 12 patients with histologically and immunohistochemically confirmed NETs were included. All cases were newly diagnosed and treatment-naïve at the time of enrolment. The study adhered to the ethical principles outlined in the Declaration of Helsinki (2013 revision) and informed consent was obtained from all participants prior to inclusion.

## Inclusion and exclusion criteria:

Patients of any age and gender presenting with primary or metastatic lesions confirmed as NETs on histopathology and immunohistochemistry were included. Only well-differentiated and moderately differentiated NETs (Grade I-III) were eligible. Cases diagnosed as NECs, mixed adenoneuroendocrine carcinomas (MANECs), or poorly differentiated neoplasms were excluded from the analysis to maintain homogeneity of tumor biology.

## Diagnostic evaluation:

All patients underwent a detailed clinical evaluation, including history, physical examination and systemic assessment of disease- related symptoms such as abdominal pain, distension, flushing, diarrhea and hypoglycemia. Tissue samples were obtained via core needle biopsy or excisional biopsy from either the primary lesion or metastatic site, depending on accessibility. The samples were processed in 10% neutral buffered formalin, embedded in paraffin and stained with hematoxylin and eosin (H & E) for histomorphological evaluation.

## Histopathological assessment:

Tumor grading was determined according to the World Health Organization (WHO) 2022 classification of gastroenteropancreatic neuroendocrine neoplasms (GEP-NENs). Grading was based on mitotic count per 10 high-power fields (HPF) and the Ki-67 proliferation index, expressed as the percentage of positively stained tumor nuclei in 500-2000 tumor cells.

## The grades were defined as follows:

[1] Grade I (Low Grade): Mitotic rate <2/10 HPF and Ki-67 ≤2%

[2] Grade II (Intermediate Grade): Mitotic rate 2-20/10 HPF or Ki-67 3-20%

[3] Grade III (High Grade): Mitotic rate >20/10 HPF or Ki-67 >20%

## Immunohistochemical (IHC) analysis:

IHC staining was performed using monoclonal antibodies against synaptophysin, chromogranin A, Ki-67 and pan-cytokeratin to confirm neuroendocrine differentiation and establish tumor grade. Additional markers, such as CDX2 and CK7/CK20, were selectively used for metastatic and site-indeterminate lesions. IHC interpretation was performed independently by two pathologists to minimize observer bias.

## Radiological and functional imaging:

All patients underwent contrast-enhanced computed tomography (CECT) or magnetic resonance imaging (MRI) for staging. 68Ga-DOTANOC PET/CT scans were performed where available to assess somatostatin receptor expression and metastatic spread. Biochemical evaluation included serum chromogranin A and 24-hour urinary 5-hydroxyindoleacetic acid (5-HIAA) estimation in functionally active cases.

## Treatment protocols:

Treatment strategies were individualized based on tumor grade, location, resectability and extent of metastatic disease.

[1] Surgical intervention was preferred for localized and resectable lesions.

[2] Somatostatin analogues (Octreotide LAR 30 mg monthly) were administered to control hormone-related symptoms in functional NETs or for palliation in metastatic cases.

[3] Chemotherapy regimens, including cisplatin plus etoposide or paclitaxel plus carboplatin, were prescribed for high-grade (Grade III) tumors.

[4] Supportive agents such as zoledronic acid were given in cases with osseous metastases.

Post-treatment follow-up included serial clinical assessments, radiologic imaging and laboratory monitoring every 3 months for the first year and every 6 months thereafter.

## Data collection and statistical analysis:

Clinical parameters including age, sex, tumor site, grade, functional status, presence of metastasis, treatment modality and survival, were recorded systematically. Descriptive statistics were applied using SPSS (version 26.0; IBM Corp., Armonk, NY). Continuous variables were summarized as mean ± standard deviation (SD) or median (range) and categorical variables as frequencies and percentages. Associations between categorical variables (e.g., tumor site vs. metastasis) were assessed using the Chi-square test, with a p-value < 0.05 considered statistically significant. Kaplan-Meier survival curves were plotted to estimate overall survival (OS) by tumor grade and intergroup differences were analyzed using the log-rank test.

## Results:

Among the 12 cases analyzed, the median age at diagnosis was 60 years (range: 17-87 years), with a male-to-female ratio of 1.4:1. Eight cases were presented with primary disease, while four had metastatic disease at the time of diagnosis. Based on the WHO 2022 classification, the distribution of primary tumor sites was as follows: NET I diagnosed in the Stomach, Appendix, Rectum, Pancreas and mesenteric Lymph node (5 cases). NET II diagnosed in the stomach (3 cases), the Duodenum (1 case) and the ileum (1 case). NET III diagnosed in the Gall bladder (1 case), Pancreas (1 case). The distribution of tumors according to the site is shown in Figure 1 (see PDF). Of 12 patients, 5 cases (41 %) presented with only functional symptoms, out of which one case had hypoglycemia in pancreatic NET, acid peptic disease in Stomach NET II, functional dyspepsia, anemia and diarrhea noted in Duodenum NET II, diarrhea and constipation alternatively seen in ileal NET grade II and Abdominal distension in Rectum NET grade I. The spectrum of clinical symptoms mentioned as tumor sites is shown in the complex bar chart in Figure 2 (see PDF).

Overall, one-third of patients (33%) presented with metastatic disease at the time of diagnosis. Gall bladder NET III tumor metastatic to the liver. Pancreatic NET III tumor metastatic to bone, lymph node, lung and liver. No statistically significant association between primary site vs metastatic presentation (Chi-Square Test p-value: 0.211), though gall bladder and pancreas show more metastasis. A 46-year-old female patient had a mesenteric growth biopsy diagnosed as NET. On CECT, evidence of DOTANOC uptake was also noted in the adjacent small bowel loops (at least 2 separate areas) with mild wall thickening (SUVmax 21.7). The liver is enlarged in size, measuring about 17.9 cm (CC). Foci of DOTANOC uptake are seen in the segment VII region of the liver. 5-HIAA - 13.28 (normal till 7). Mesenteric growth, which possibly originated from mid bowel growth, was diagnosed as NET grade II by histomorphology. IHC was not performed due to scanty tissue. Two patients were associated with other solid malignancies, out of which one case details that a 74-year-old female diagnosed with endometroid carcinoma grade III underwent total abdominal hysterectomy with bilateral salpingectomy, bilateral pelvic and retroperitoneal lymphadenectomy, along with supracolic omentectomy. One of the retroperitoneal lymph nodes revealed features of a neuroendocrine tumor grade 1, which was confirmed by immunohistochemistry. The patient is lost to follow-up; further work-up has not been done. Another patient, F/56 year, presented with symptoms of bleeding per vagina, abdominal pain, weight loss and loss of appetite for 3 months. Diagnosed as High-grade serous carcinoma of the ovary and received chemotherapy with Paclitaxel + Carboplatin. An Upper Endoscopy was performed to evaluate the chronic liver disease, revealing oesophageal varices and small nodules at the proximal body along the greater curvature of the stomach. A biopsy was taken from the nodule in the stomach and the histological findings favour a NET G1 tumor. IHC markers were positive for synaptophysin and chromogranin. MIB 1 index was 3-4% in foci, suggesting the coexistence of two tumors at two distinct locations, appearing sequentially. Out of 12 patients, 5 (41.7%) had Grade 1 (low grade), 5 (41.7%) had Grade 2 (intermediate grade) and 2 (16.3%) had Grade 3 (high grade). GEP-NET I and II tumors were found in the stomach, duodenum, rectum, appendix, ileum, mesentery, while grade III tumors were observed in the pancreas and gall bladder. The histopathological images of some representative cases are illustrated in Figure 3 (see PDF). The cells of the liver tumor were IHC positive for CDX2, Synaptophysin, Cytokeratin - Positive Ki 67 -20-25%, suggestive of NET grade III.

Stomach growth (H and E X400) - shows acini and cords pattern of epithelial cells with monomorphic nuclei, pepper salt chromatin and a moderate amount of cytoplasm. Mitotic activity 1-2/10 HPF. Favor of NET Grade 1 with tumor component; C) Duodenal growth (H and E X100) - shows duodenal mucosa with tumor component; D) Duodenal growth (H and E X400) -Shows a discohesive sheet of epithelial cells, dispersed chromatin and scanty cytoplasm. Mitotic activity - 3-5/10 HPF; E) Liver biopsy (H and E X100)- shows scanty liver parenchyma with more tumor component. Imp- Metastatic tumor deposits from Neuroendocrine tumor origin; F) Liver biopsy (Hand E X400) - shows mostly tumor component, consisting of a discohesive sheet of epithelial cells with enlarged nuclei, moderate amount of cytoplasm and increased mitotic activity. Although not statistically significant, metastatic disease at presentation was the most frequently associated with grade III NETs (76.5%) as compared to Grade 1 (51.6%) and Grade 2 (68.75%), indicating a trend toward higher metastatic potential in advanced grades. NET III demonstrated a wide Ki 67 index range (25 - 80 %), with a notably high proportion (80%) showing elevated Ki 67 scores. However, no statistically significant correlation was found between tumor grade and tumor site. Of 12 patients, one patient with endometrial carcinoma grade III and mesenteric lymph node NET grade I did not turn up for definitive treatment planning and was considered lost to follow-up. The remaining nine patients diagnosed with NET grade I and II were managed primarily with surgery and observation, except for one case of Ileal NET grade II, which was treated with octreotide and zoledronic acid. The patient with Pancreatic NET grade III received carboplatin weekly, zoledronic acid every four weeks and octreotide LAR 30 mg. The patient with gall bladder NET grade III was treated with 3-4 cycles of cisplatin plus etoposide, followed by three cycles of paclitaxel plus carboplatin. A total of three patients underwent curative surgical resection. Including one distal pancreatectomy and two gastrectomies. All achieved, complete (R0) resection with a 100 % surgical complete response (CR) regardless of NET grade. Ten patients with Grade I and II NETs were placed under close observation, except for one metastatic case managed with octreotide LAR 30 mg for symptom relief. Overall survival analysis was performed for 11 patients. NET I and II tumors showed 100 % survival, whereas NET III tumors had a 50% survival rate, with a median survival of 15 months (Table 1 - see PDF).

## Discussion:

This study contributes to the limited data on NETs in the Indian subcontinent by evaluating their clinicopathological spectrum, treatment responses and survival outcomes using the updated WHO 2022 Endocrine and Neuroendocrine Neoplasm classification. A Western study reported a higher median age at presentation of neuroendocrine tumors above 60 years, with a female predominance [7]. Recent evaluations from Norway (2017-2021) indicate the most common NET localizations were the small intestine (23%) and the lungs (19%) [8] and the small intestine and rectum in the Taiwanese study [9]. In contrast, a study from Western India by Kulkarni *et al.* [10] reported a median age of 49 years and male predominance, with the pancreas being the most frequent primary site (35%). The stomach and pancreas (32.5 % cases) were the most frequently involved sites in the study by Kapoor *et al.* In our study, the median age at presentation was 63 years, similar to Western literature. A male predominance was also observed, consistent with findings of the western India study. Stomach (33.3 %) emerged as the most frequently involved site, in agreement with observation by Kapoor *et al.* Functional NETs were more common (41%) than previously reported in Indian studies (~4%) [11] But aligned with international data (20-30%) [12]. The metastatic disease at presentation observed in our study was 33%, according to the primary sites originating from the gall bladder, pancreas and ileum. The metastatic presentation falls within the range reported globally (21-53%) [11, 12]. Hallett *et al.* reported a total of 5,619 NET cases, with 20.8% presenting with metastasis [13]. In Kulkarni *et al.*'s study [10], metastatic disease was present at diagnosis in about 66 % of cases. The pattern of initial presentation (metastatic vs. non-metastatic) varied by the site of origin of NETs, with metastasis being observed in the vast majority (100%) in tumors of the large intestine, gallbladder, breast and rectum (80%), as well as the stomach (75%). No statistically significant association was observed between primary site and metastatic presentation, likely due to the small sample size. However, the gall bladder and pancreas demonstrated a greater tendency to present with metastasis. In our study, one case of NET grade II in the stomach was associated with endometroid carcinoma of the uterus and another case was diagnosed as High-grade serous carcinoma of the ovary with NET grade I deposits in the retroperitoneal lymph node. This rare finding shows a lack of evidence compared to other studies. The disease progression of NET grade III tumors was more invasive than that of grade II tumors, but a more favorable course than poorly differentiated NECs. According to findings by Velayoudom-Cephise *et al.* well- differentiated grade III NETs account for 5- 8% of GEP-NETs in different studies [14]. In our cohort, Grade III NETs represented 18% of cases (primarily in the pancreas and gallbladder), which is higher than global reports and these patients required systemic therapy. Response to platinum and etoposide chemotherapy has been reported between 47.4% to 68% [15]. Our study also showed promising efficacy in high-grade (G3) NETs. In line with previous evidence, somatostatin analogues provided symptom control in G1/G2 NETs; no measurable tumor regression was observed [16]. In our cohort, one patient with metastatic NET received intramuscular octreotide LAR 30 mg for symptomatic relief. Well-differentiated NETs are confirmed by positive staining for synaptophysin and chromogranin, with grading assessed using Ki-67. Additional markers may aid in determining the primary site, such as CDX2 for bowel, TTF1 for lung and PAX8, ISL1 and PDX1 for pancreas [17]. The distinction between pancreatic NEC and pancreatic NET is driven by unique molecular pathogenetic alterations, with NEC characterized by global loss of RB and aberrant p53, while NET more often demonstrates loss of ATRX, DAXX, or Menin along with differential expression of SSTRs [18]. In our study, a molecular analysis was not conducted on NET Grade III tumors. Future directions include the development of molecular biomarkers (e.g., p53, RB, ATRX, DAXX) to differentiate G3 NETs from NECs and guide targeted therapies. The limited sample size reduced the statistical strength of the study. Post-treatment surveillance and multicentre research are vital to acquire a clearer understanding of the biological behaviour of NET in the Indian population.

## Conclusion:

NETs are a diverse group of tumors with significant clinical challenges due to variable behaviour, often late presentation and are very challenging to diagnose, especially NET grade III. It must be differentiated from NECs using IHC markers. A significant proportion of patients in our study had functional NETs and presented with metastatic disease, highlighting the need for early detection protocols. Further multi-institutional studies are needed to more clearly establish the epidemiology, clinical presentation, treatment strategy and prognosis of this "low-prevalence" yet not truly rare disease.
